# Global research hotspots and trends of theta burst stimulation from 2004 to 2023: a bibliometric analysis

**DOI:** 10.3389/fneur.2024.1469877

**Published:** 2024-12-10

**Authors:** Mingyue Liu, Shasha Jin, Mengya Liu, Bin Yang, Qian Wang, Chunliang Fan, Zhe Li, Liang Wu

**Affiliations:** ^1^Department of Sports Rehabilitation, Beijing Xiaotangshan Hospital, Beijing, China; ^2^Department of Rehabilitation Medicine, The Fifth Affiliated Hospital of Zhengzhou University, Zhengzhou, China; ^3^Department of Rehabilitation Medicine, The First Affiliated Hospital of Xi’an Jiaotong University, Xi’an, China; ^4^Department of Physical Therapy, Beijing Xiaotangshan Hospital, Beijing, China

**Keywords:** theta burst stimulation, bibliometric analysis, hotspots and trends, VOSviewer, CiteSpace

## Abstract

**Background:**

Theta burst stimulation (TBS) has garnered widespread attention in the scientific community, but a comprehensive bibliometric analysis of TBS research remains absent. This study aims to fill this gap by elucidating the characteristics, hotspots, and trends in TBS publications over the past 20 years using bibliometric methods.

**Methods:**

We retrieved TBS-related publications from January 1, 2004, to December 31, 2023, from the Web of Science Core Collection (WoSCC). The analysis focused on articles and review articles. Data were processed using the bibliometric package in R software, and CiteSpace and VOSviewer were employed for bibliometric and knowledge mapping analyses.

**Results:**

A total of 1,206 publications were identified, with 858 included in the analysis. The annual publication volume showed a fluctuating upward trend. Leading institutions and authors were predominantly from the United States of America (USA) and European countries. Core journals and publications also primarily originated from these regions. Current research hotspots include the clinical applications and mechanisms of TBS in neurorehabilitation and depression. TBS cerebellar stimulation has emerged as a promising therapeutic target. Future research is likely to focus on dysphagia, cognitive impairments, and post-traumatic stress disorder.

**Conclusion:**

This bibliometric analysis provides an overview of the basic knowledge structure, research hotspots, and development trends in TBS research over the past two decades. The findings offer valuable insights into the evolving landscape of TBS research and its potential directions.

## 1 Introduction

Endogenous theta frequency oscillations in hippocampal and cortical circuits are critical for learning, motor function, and memory processing (1). Theta burst stimulation (TBS) mimics this natural electrophysiological activity, providing a unique non-invasive neural stimulation method (2). The most commonly used TBS paradigms include intermittent TBS (iTBS) and continuous TBS (cTBS), each modulating cortical excitability through distinct stimulation patterns. The iTBS protocol consists of 2 s of continuous stimulation followed by an 8-s interval, repeated in cycles, and is believed to induce long-term potentiation (LTP) (3). In contrast, cTBS involves uninterrupted bursts of stimulation at a fixed frequency for 40 to 50 s, which is thought to induce long-term depression (LTD) (4). LTP and LTD are fundamental concepts in synaptic plasticity, considered key mechanisms underlying learning and memory. However, the manifestation and functional role of these mechanisms in the human brain remain contentious, as most human studies on LTP and LTD are extrapolated from animal models (5, 6). Some research suggests that other forms of synaptic plasticity, such as short-term plasticity and synaptic normalization mechanisms, may also play significant roles in learning and memory, potentially interacting with LTP and LTD in a collaborative manner (7). As a form of patterned repetitive transcranial magnetic stimulation (rTMS), TBS offers several advantages over traditional rTMS, including shorter stimulation times, lower intensity, longer-lasting effects, and a stimulation pattern that more closely resembles natural neural activity (8). These characteristics not only enhance the safety and comfort of TBS but also improve its specificity and efficacy in modulating neural network functions (9). Therefore, TBS holds significant potential in both basic neuroscience research and clinical applications, providing new perspectives on the regulation of brain function (10–13).

Despite extensive research into TBS, its diverse and complex research directions present challenges for newcomers and researchers in the field. Bibliometrics, an interdisciplinary field that applies mathematical and statistical methods to analyze written communication, can provide valuable insights into the quantitative aspects of literature, including publication volume, citation impact, and spatial distribution. This method reveals the development status and trends within a field, helping to identify academic frontiers, hotspots, and evolving research themes (14). Bibliometric analysis has widespread applications in academic research, discipline development, scientific evaluation, and information services (15, 16). However, to date, no comprehensive bibliometric analysis has been conducted specifically on TBS research. The systematic knowledge structure, evolutionary paths, and research hotspots in this field remain underexplored. This study aims to fill this gap by using bibliometric methods to analyze TBS-related publications from the Web of Science Core Collection (WoSCC) over the past 20 years. Our goal is to visually present the research framework, identify key trends, and explore the evolving hotspots in TBS research, thereby offering valuable insights for future investigations in this rapidly developing field.

## 2 Materials and methods

### 2.1 Data sources and search strategy

**Figure 1:**
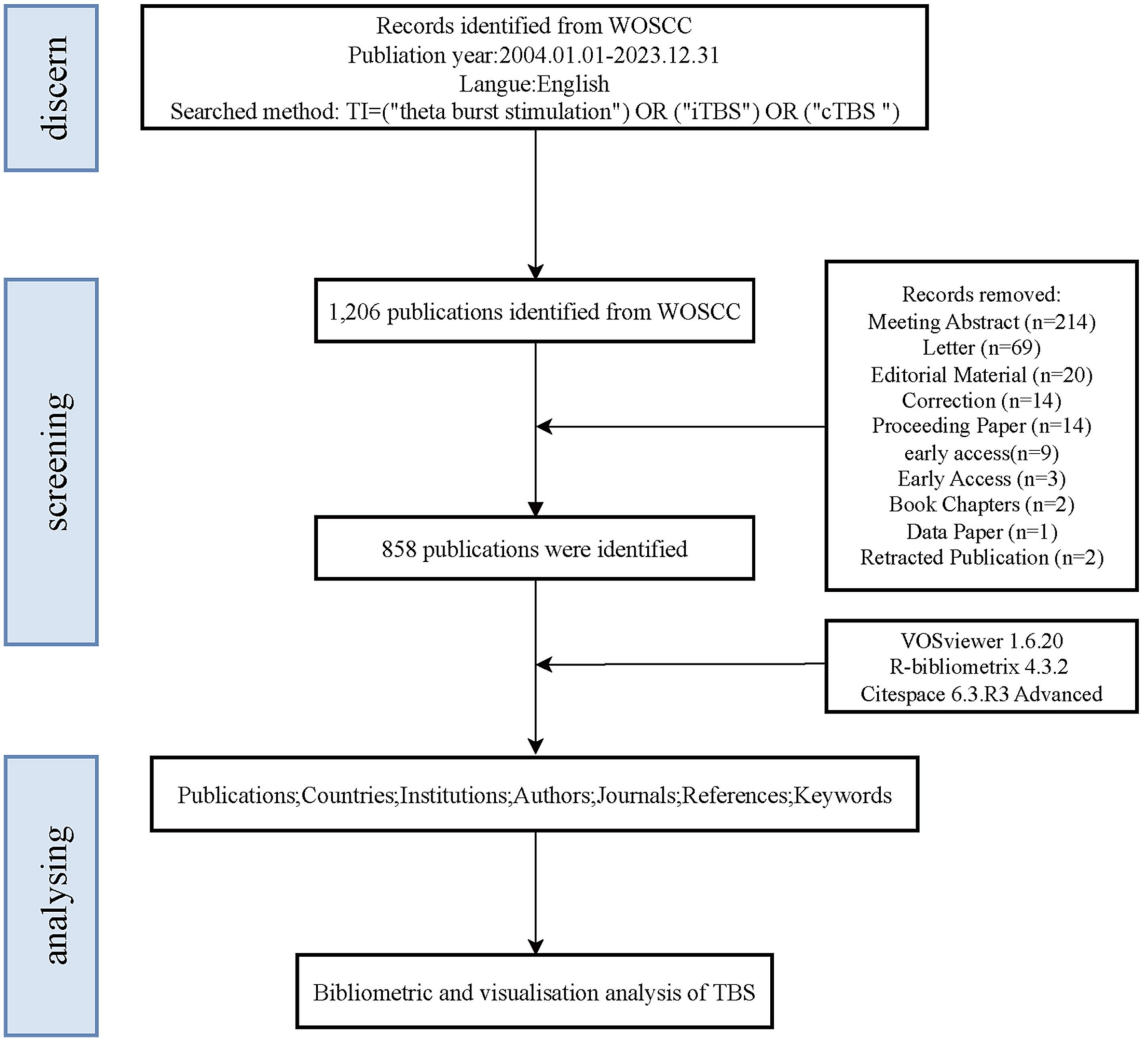
Selection and bibliometric analysis workflow of TBS-related research publications.

Given that the Web of Science Core Collection (WoSCC) and Scopus are widely recognized as the leading bibliometric databases, other databases that do not provide co-citation data significantly limit the scope and depth of bibliometric analyses (17). Although Scopus is a comprehensive resource, it includes a substantial number of articles without impact factors, which may introduce a degree of uncertainty regarding the reliability of the analytical results (18). To ensure a robust and systematic analysis, this study utilized the WoSCC. The WoSCC encompasses the following sub-databases: the Science Citation Index Expanded (SCI-EXPANDED), the Social Sciences Citation Index (SSCI), the Arts & Humanities Citation Index (A&HCI), the Conference Proceedings Citation Index—Science (CPCI-S), the Emerging Sources Citation Index (ESCI), the Current Chemical Reactions Index (CCR-EXPANDED), and the Index Chemicus (IC). A title-based search was conducted on June 1, 2024, using the query: TI = (“theta burst stimulation”) OR (“iTBS”) OR (“cTBS”) to identify relevant publications from January 1, 2004, to December 31, 2023. Given the relatively limited number of publications in the field of TBS prior to 2004, this timeframe was deemed appropriate to represent the current state of research in this area. The inclusion criteria were restricted to articles and reviews published in English. Following independent searches conducted by two researchers, and subsequent cross-verification, non-relevant publications—such as letters, newspapers, conference papers, and news articles—that did not meet the inclusion criteria were excluded. Duplicate records were also removed. Ultimately, a total of 858 publications were included in the analysis. Relevant documents were exported in TXT format, which included full-text records and references. These data were then imported into bibliometric analysis software for subsequent visualization. The study workflow is illustrated in Figure 1.

### 2.2 Data processing and analysis

For the comprehensive quantitative analysis of publication volume, countries, institutions, authors, journals, references, and keywords, we employed CiteSpace (version 6.1.R3 Advanced), VOSviewer (version 1.6.20), and the R-bibliometrix package (version 4.3.2) (19) (detailed variable analysis is provided in Supplementary material S1). Prior to the analysis, several preprocessing steps were implemented to ensure data quality. These steps included the normalization of synonyms, removal of irrelevant terms, and standardization of variations in author and institutional names (specific preprocessing details are provided in Supplementary material S2). Using CiteSpace, we extracted detailed information from the data, including collaboration networks among countries and institutions, trends in disciplinary development, citation and co-citation analyses, and the identification of emerging research trends (20) (see Supplementary material S3 for detailed visual interpretations). VOSviewer facilitated the extraction and visualization of key insights from the publication data, particularly through the construction of co-occurrence networks of keywords, which revealed the structure and dynamics of scientific research (21). R-bibliometrix, an open-source tool within the R environment, generated various visual outputs, such as cooperation and trend graphs, thereby enabling the intuitive presentation of the analytical results (22). By integrating the functionalities of these tools, we produced co-occurrence, clustering, and highlighting maps that provide a multi-dimensional view of the TBS field, thereby supporting corresponding analyses.

## 3 Results

### 3.1 Annual publication and citation growth trend

**Figure 2:**
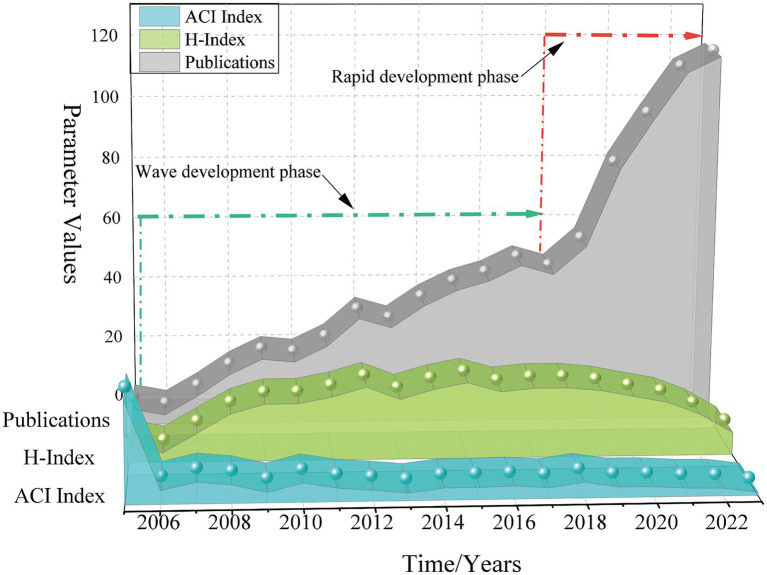
Evolution trend of the quantity and citation of publications related to TBS research.

Based on the research strategy outlined, a total of 858 publications related to TBS were retrieved from the WoSCC database for the period 2004–2023. The annual number of publications (Np), the average citations per article (ACI), and the Hirsch index (*H*-index) are presented in Figure 2. No related publications were recorded in 2004. From 2005 to 2009, the number of publications grew slowly and steadily, with a slight stagnation observed in 2010, followed by a rapid increase after 2018. The *H*-index of publications from 2004 to 2010 gradually increased, remained stable from 2010 to 2018, and declined post-2019 due to time constraints. The ACI was relatively high between 2005 and 2008, with the highest value observed in 2005.

### 3.2 Analysis of countries/regions

**Figure 3:**
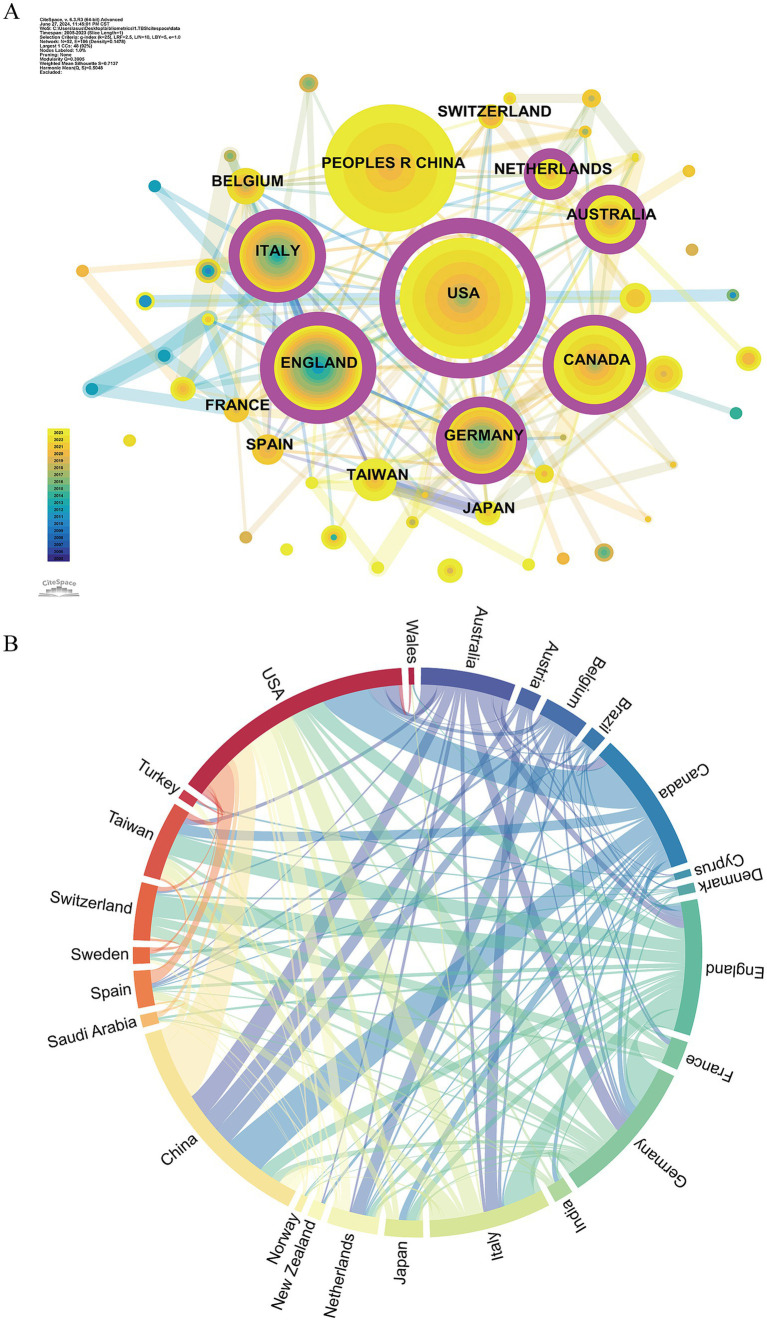
The national/regional analysis of TBS-related research. **(A)** The co-occurrence country map of TBS research. Node size indicates co-occurrence frequency, with purple circles representing high Bc (≥0.1). **(B)** The network graph illustrating publication output and collaboration between countries/regions.

A total of 57 countries/regions have contributed to TBS-related research. Statistics for the top 10 countries/regions, based on the number of TBS publications, are presented in Table 1. The USA (Np: 180) and China (Np: 161) are the leading contributors, followed by Germany, Canada, and other regions with fewer than 100 publications each. Notably, while the USA and China together account for nearly 40% of the publications in the TBS field, the number of citations (Nc) for the USA was 4,066, which is 2.7 times greater than that of China (Nc: 1,499). The USA also exhibited the highest betweenness centrality (Bc: 0.6), indicating its dominant influence in terms of both the quantity and quality of publications in this field. In the country co-occurrence map (Figure 3A), purple circular nodes represent countries with high Bc (≥0.1). The top five countries by Bc are the USA, the United Kingdom (UK), Germany, Australia, and Canada. Figure 3B illustrates the strong international collaboration, with the most frequent partnerships occurring between the USA and the UK, followed by collaborations between the USA and Germany.

**Table 1:** The top 10 countries/regions by production of TBS-related publications (WoS).

Rank	Country/region	Np	Bc	Nc	*H*-index	ACI
1	USA	180	0.60	4,066	34	22.59
2	China	161	0.01	1,499	21	9.31
3	Germany	105	0.18	3,531	33	33.63
4	Canada	102	0.15	2,803	27	27.48
5	UK	99	0.24	8,114	41	81.96
6	Italy	89	0.14	3,844	35	43.19
7	Australia	68	0.16	2,410	29	35.44
8	Taiwan	56	0.04	5,249	24	93.73
9	Switzerland	42	0.01	1,341	22	31.93
10	Netherlands	37	0.10	875	17	23.65

### 3.3 Analysis of institutions

**Figure 4:**
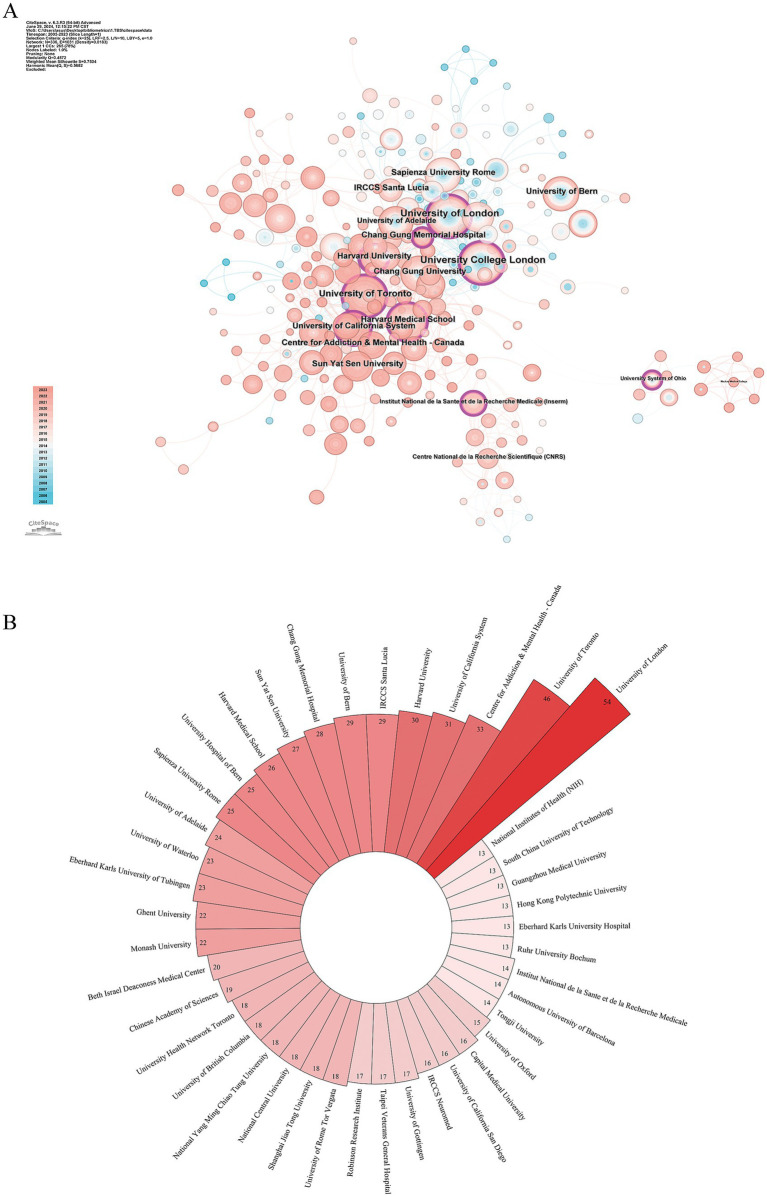
The institutional analysis of TBS-related research. **(A)** Co-occurrence institutional map of TBS research. **(B)** South-Nightingale rose diagram showing institutional publication output.

Figure 4A presents the co-occurrence network of major research institutions, with detailed information on the top 10 institutions by publication volume shown in Table 2. Figure 4B displays a Nightingale rose chart representing the overall publication volume. In terms of the number of publications, the University of London (UK) contributed the most (Np: 54), followed by the University of Toronto (Canada) with 46 publications and the Centre for Addiction and Mental Health (Canada) with 33 publications. In terms of Bc, the University of London, the University of California, and Harvard University (USA) ranked in the top three. Institutions with high Bc demonstrate close collaboration, indicating a strong scientific capability in TBS research.

**Table 2:** The top 10 institutions by production of TBS-related publications (WoS).

Rank	Institution	Country/region	Np	Bc
1	University of London	UK	54	0.19
2	University of Toronto	Canada	46	0.08
3	Centre for Addiction & Mental Health-Canada	Canada	33	0.03
4	University of California	USA	31	0.17
5	Harvard University	USA	30	0.12
6	IRCCS Santa Lucia	Italy	29	0.08
7	University of Bern	Switzerland	29	0.02
8	Chang Gung Memorial Hospital	Taiwan	28	0.07
9	Sun Yat-sen University	China	27	0.07
10	University Hospital of Bern	Switzerland	25	0.01
11	Sapienza University Rome	Italy	25	0.06

### 3.4 Analysis of authors

**Figure 5:**
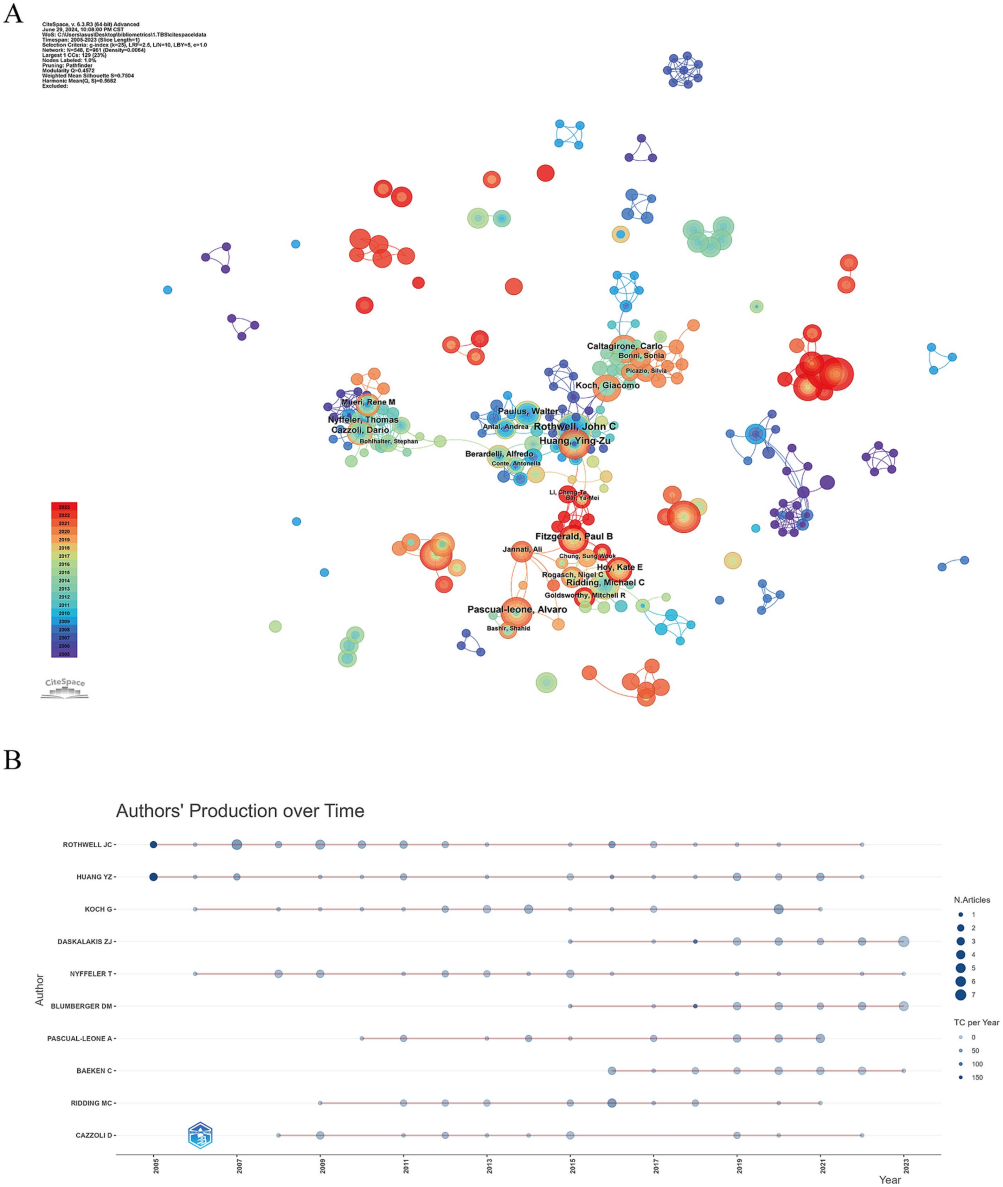
The author analysis of TBS-related research. **(A)** Co-occurrence authorship map of TBS research. **(B)** Annual publication trend of prolific authors.

Price’s Law was applied to calculate the minimum publication volume of core authors using the mathematical model $M\approx 0.749\times \sqrt{n_{\text{max}}}$ , where M represents the minimum number of publications for core authors, and $n_{\text{max}}$ is the highest publication count by a single author. Analysis using CiteSpace software identified $n_{\text{max}}=25$ , leading to M≈3.745 . Therefore, authors with four or more publications are classified as core authors, totaling 190 authors (4.96% of all authors). Based on CiteSpace data, a ranking table of the top 10 authors was constructed (Table 3). John C. Rothwell from the Institute of Neurology at University College London leads with 25 publications, followed by Zafiris J. Daskalakis from the University of Toronto and Ying-Zu Huang from Chang Gung University College of Medicine, each with 21 publications. In the co-occurrence network of core authors, most scholars are associated with their own research teams, demonstrating close internal cooperation but a lack of prominent high-Bc authors (Figure 5A). The trend graph of annual publication volumes of high-output authors (Figure 5B) highlights recent active authors in this field, including Zafiris J. Daskalakis, Daniel M. Blumberger, and Chris Baeken. These authors exhibit significant academic vitality, and their research outputs warrant further attention.

**Table 3:** The top 10 authors by production of TBS-related publications (WoS).

Rank	Author	Np	Bc	Nc	ACI
1	Rothwell, John C.	25	0.05	1,704	68.16
2	Daskalakis, Zafiris J.	21	0	283	13.48
3	Huang, Ying-Zu	21	0.01	1,400	66.67
4	Koch, Giacomo	20	0.01	242	12.10
5	Nyffeler, Thomas	19	0	261	13.74
6	Blumberger, Daniel M.	19	0	269	14.16
7	Baeken, Chris	18	0	104	5.78
8	Pascual-leone, Alvaro	18	0.02	236	13.11
9	Ridding, Michael C.	16	0	359	22.44
10	Fitzgerald, Paul B.	16	0	317	19.8

### 3.5 Analysis of journal

**Figure 6:**
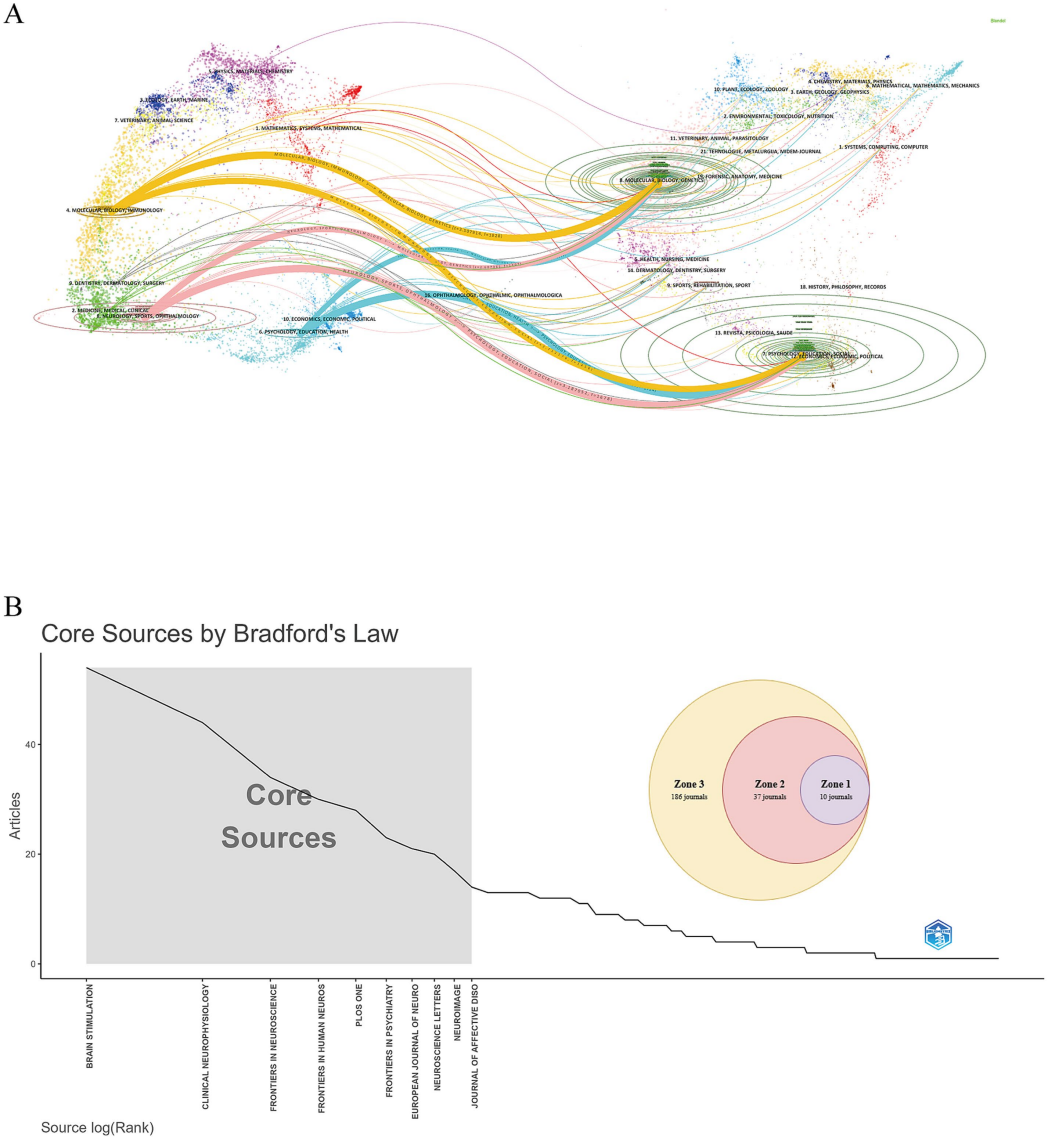
The journal analysis of TBS-related research. **(A)** Double-map overlay of TBS research journals. The left side depicts citing journal clusters, while the right side depicts cited journals, with colored trajectories indicating citation relationships. **(B)** Grouping of academic journals based on Bradford’s Law.

During the study period, TBS-related publications appeared in 236 journals. Table 4 shows that the journal with the most publications is *Brain Stimulation* (Np: 54), followed by *Clinical Neurophysiology* (Np: 44), *Frontiers in Neuroscience* (Np: 34), and *Frontiers in Human Neuroscience* (Np: 30). Figure 6A presents a dual-map overlay of journals, visually representing journal distribution, citation patterns, and shifts in research focus. In the TBS field, journals in the categories of molecular biology/immunology and neurology/sports science/ophthalmology frequently cite articles published in journals within the fields of molecular biology/genetics. Figure 6B shows the results of grouping journals according to Bradford’s Law, with the core zone (Zone 1) comprising 10 journals, the secondary core zone (Zone 2) consisting of 37 journals, and the non-core zone (Zone 3) containing 186 journals.

**Table 4:** The top 10 journals publishing TBS-related publications (WoS).

Rank	Source	Country	Np	Nc	ACI	Impact factor (2023)
1	Brain Stimulation	USA	54	2,493	46.17	7.6
2	Clinical Neurophysiology	Netherlands	44	2,420	55.00	3.7
3	Frontiers in Neuroscience	Switzerland	34	397	11.68	3.2
4	Frontiers in Human Neuroscience	Switzerland	30	303	10.10	2.4
5	PLoS One	USA	28	594	21.21	2.9
6	Frontiers in Psychiatry	Switzerland	23	98	4.26	3.2
7	European Journal of Neuroscience	UK	21	827	39.38	2.7
8	Neuroscience Letters	Netherlands	20	454	22.70	2.5
9	NeuroImage	USA	17	517	30.41	4.7
10	Journal of Affective Disorders	Netherlands	15	318	21.20	4.9

### 3.6 Analysis of reference

Based on Table 5, the top 10 most cited publications on TBS are presented, with the top three each cited over 300 times. The most cited publication is “*Theta Burst Stimulation of the Human Motor Cortex*” by Huang Y. Z. et al., published in *Neuron* in 2005, which has been cited 2,758 times. This pivotal study introduced the definition of TBS, providing a foundation for subsequent research in the field. The second most cited work is “*Effectiveness of Theta Burst* versus *High-Frequency Repetitive Transcranial Magnetic Stimulation in Patients with Depression (THREE-D): A Randomised Non-Inferiority Trial*” by Blumberger D. M. et al., published in *The Lancet* in 2018, with 609 citations. The top 10 references cover a broad spectrum of topics, including neurophysiological mechanisms, clinical applications, and the optimization of TBS protocols.

**Table 5:** The top 10 most-cited references on TBS (WoS).

Rank	Title	First author	Year	Journal	Nc
1	Theta burst stimulation of the human motor cortex	Huang Y. Z.	2005	Neuron	2,758
2	Effectiveness of theta burst versus high-frequency repetitive transcranial magnetic stimulation in patients with depression (THREE-D): a randomised non-inferiority trial	Blumberger D. M.	2018	Lancet	609
3	The after-effect of human theta burst stimulation is NMDA receptor dependent	Huang Y. Z.	2007	Clinical Neurophysiology	431
4	Ten years of theta burst stimulation in humans: established knowledge, unknowns and prospects	Suppa A.	2016	Brain Stimulation	341
5	Theta-burst repetitive transcranial magnetic stimulation suppresses specific excitatory circuits in the human motor cortex	Di Lazzaro V.	2005	Journal of Physiology	285
6	Depression of human corticospinal excitability induced by magnetic theta-burst stimulation: evidence of rapid polarity-reversing metaplasticity	Gentner R.	2008	Cerebral Cortex	282
7	Theta burst stimulation dissociates attention and action updating in human inferior frontal cortex	Verbruggen F.	2010	Proceedings of the National Academy of Sciences of the United States of America	259
8	Theta-burst transcranial magnetic stimulation to the prefrontal cortex impairs metacognitive visual awareness	Rounis E.	2010	Cognitive Neuroscience	258
9	The physiological basis of the effects of intermittent theta burst stimulation of the human motor cortex	Di Lazzaro V.	2008	Journal of Physiology	241
10	Simply longer is not better: reversal of theta burst after-effect with prolonged stimulation	Gamboa O. L.	2010	Experimental Brain Research	225

**Figure 7:**
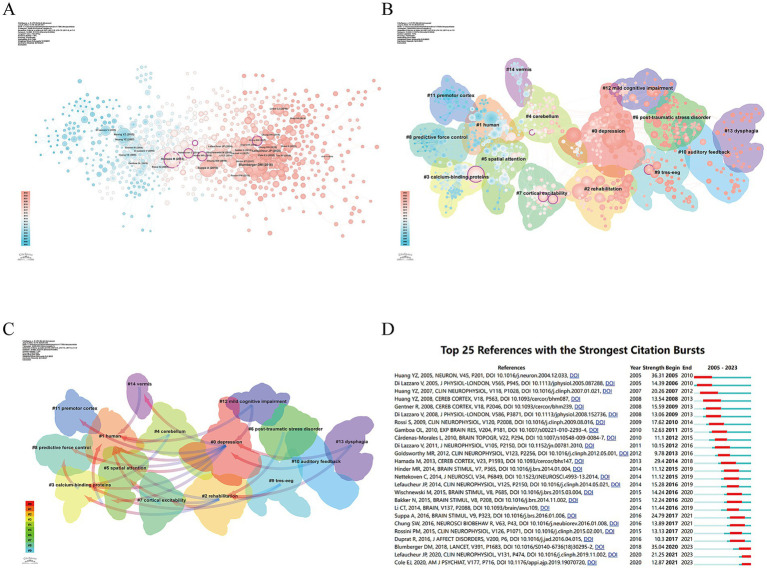
The reference analysis of TBS-related research. **(A)** Co-citation relationship map among references. **(B)** Clustering of references based on similarity, including 15 main clusters such as #0 depression, #1 human, #2 rehabilitation, etc. **(C)** Dependency analysis between literature clusters, reflecting the evolution process among them. **(D)** Top 25 references with the highest citation bursts, indicated by red lines in corresponding years.

In Figure 7A, studies with higher Bc predominantly focus on the application and potential of TBS in areas such as neurophysiological and pathological mechanisms, as well as neurosurgical rehabilitation. The cluster analysis of references (Figure 7B) provides an objective view of the knowledge structure in TBS research. The references are categorized into 15 clusters based on the degree of correlation between publications. The largest cluster, #0 focuses on depression, while earlier research clusters include #3 calcium-binding proteins, #8 predictive force control, #11 premotor cortex, and #14 vermis. Subsequent studies have evolved into clusters focused on #0 depression and #2 rehabilitation. In recent years, however, the connectivity between research fields has decreased, with clusters such as #6 post-traumatic stress disorder, #9 transcranial magnetic stimulation combined with electroencephalography (TMS-EEG), #10 auditory feedback, #12 mild cognitive impairment, and #13 dysphagia becoming more independent. These clusters reflect a growing focus on the extension and refinement of TBS applications in clinical disease-related syndromes.

Dependency analysis of the reference clusters, conducted using CiteSpace (Figure 7C), provides a clearer understanding of the current research hotspots and the evolutionary relationships among these clusters. The clusters can be categorized into three main groups: foundational research, bridging clusters, and frontier clusters. Foundational research clusters refer to those that have evolved into other clusters, such as Cluster #11 premotor cortex, Cluster #8 predictive force control, Cluster #1 human, and Cluster #5 spatial attention. Bridging clusters, on the other hand, are those that have both evolved from other clusters and subsequently given rise to additional clusters, serving a connective role in the research process. These include Cluster #3 calcium-binding proteins, Cluster #14 vermis, Cluster #7 cortical excitability, Cluster #2 rehabilitation, and Cluster #0 depression. Among these, Cluster #0 depression stands out with the highest link strength, indicating its significant research prominence. It has evolved from Clusters #14 vermis, #11 premotor cortex, #8 predictive force control, #5 spatial attention, #3 calcium-binding proteins, and #1 human, and further evolved into Clusters #12 mild cognitive impairment, #13 dysphagia, and #10 auditory feedback. A closely related cluster, Cluster #2 rehabilitation, evolved from Clusters #1 human, #3 calcium-binding proteins, #5 spatial attention, and #7 cortical excitability, and subsequently evolved into Clusters #6 post-traumatic stress disorder, #9 TMS-EEG, and #13 dysphagia. Finally, Clusters #6 post-traumatic stress disorder, #9 TMS-EEG, #10 auditory feedback, #12 mild cognitive impairment, and #13 dysphagia have evolved from other clusters but have not yet evolved into additional clusters, suggesting that these topics likely represent the frontier areas of TBS research in recent years.

Figure 7D displays the top 25 references with citation bursts, where the burst duration exceeds 2 years, and the average burst strength is over 9 years, indicating their significant academic contribution. The strongest burst (strength = 36.31) occurred for the groundbreaking 2005 study by Huang Y. Z. et al. In the past 5 years, the study by Blumberger D. M. et al., published in *The Lancet* in 2018, has become highly prominent.

### 3.7 Analysis of keyword

**Figure 8:**
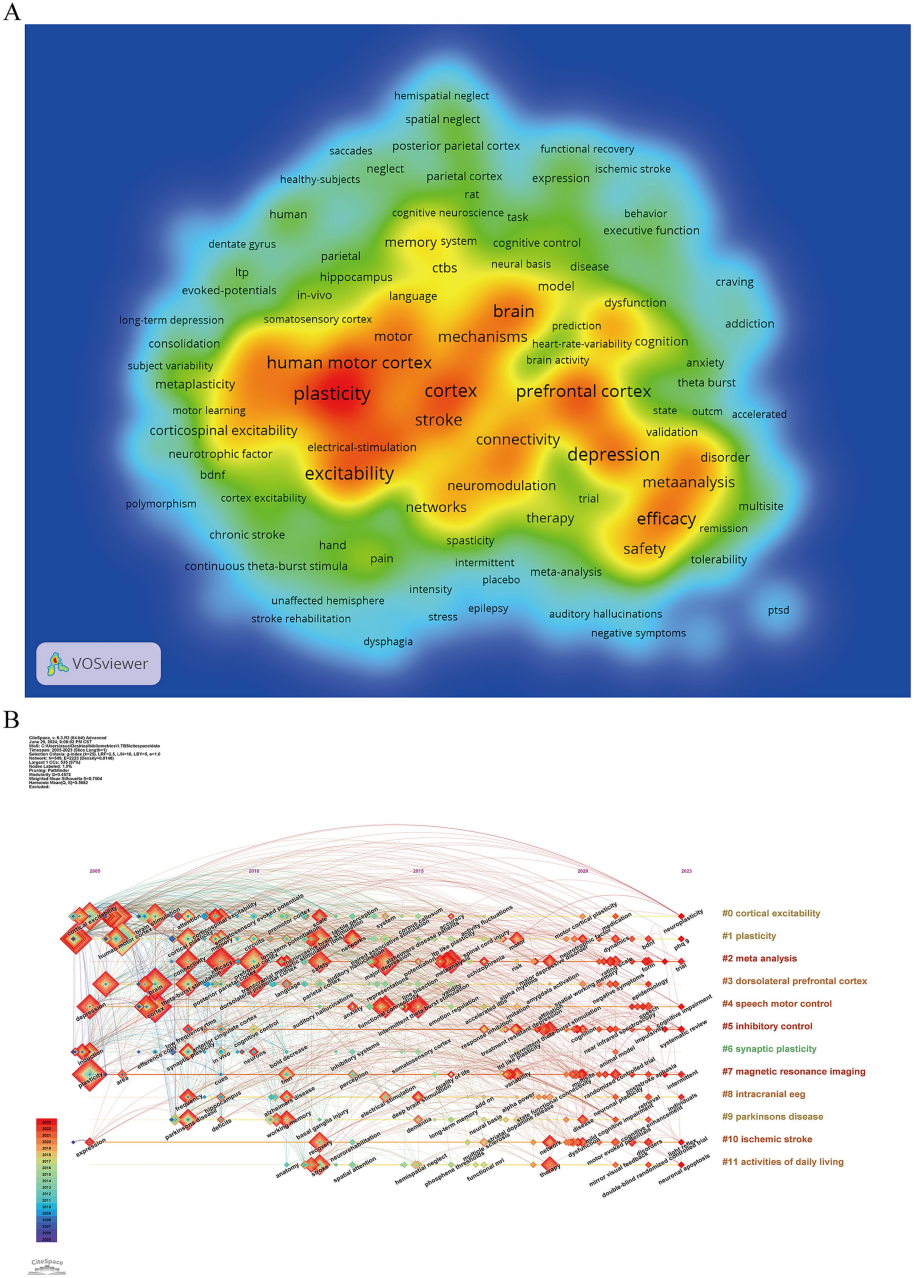
The keyword analysis of TBS-related research. **(A)** Co-occurrence network of keywords in TBS research. **(B)** Timeline view of keyword clusters.

The keyword density map in Figure 8A highlights the primary research focal points within the TBS field. Prominent terms such as *plasticity*, *depression*, *excitability*, *motor cortex*, *prefrontal cortex*, *efficacy*, and *stroke* frequently appear, each occurring over 80 times with a link strength exceeding 500. These keywords are primarily associated with investigations into the neurophysiological mechanisms of TBS and its clinical applications in specific diseases and symptoms.

Figure 8B presents both a cluster map and a timeline view of TBS-related keywords, illustrating the temporal distribution of prominent research topics. Cluster #0 cortical excitability stands out as the largest and most frequently cited research hotspot, having evolved into a major area of focus. Alongside Cluster #0, Clusters #1 plasticity, #2 meta-analysis, #3 dorsolateral prefrontal cortex, #4 speech motor control, #7 magnetic resonance imaging, and #10 ischemic stroke exhibit extended time spans, representing sustained areas of interest in TBS research. In contrast, clusters #5 inhibitory control, #8 intracranial electroencephalography, #9 Parkinson’s disease, and #11 activities of daily living have emerged as relatively new areas of investigation. Furthermore, certain keywords, such as cortical excitability and plasticity, have remained consistent throughout the course of research, while others, such as meta-analysis and near-infrared spectroscopy, have only gained significant attention in recent years. Overall, the evolution of these key research terms reflects an initial focus on TBS paradigms and neurophysiological mechanisms, whereas contemporary research has increasingly centered on TBS applications across various diseases and symptoms, as well as an exploration of the central mechanisms involved, particularly within the framework of multimodal diagnostic tools.

## 4 Discussion

This study employs bibliometric analysis to provide a comprehensive examination of the development and key trends in TBS research, outlining two distinct phases of its progression: the exploratory phase from 2005 to 2018, and the period of rapid growth from 2018 to the present. The analysis investigates several critical metrics, including publication volume, collaboration networks, and citation frequencies, offering a clear view of the global distribution and influence of TBS research. Notably, leading countries, prominent institutions, and key authors have played a pivotal role in shaping the field. However, challenges remain in promoting international collaboration and enhancing academic diversity. Furthermore, the focus of TBS research has gradually shifted from investigating neurophysiological mechanisms to exploring its clinical applications, particularly in neurological rehabilitation and mental health disorders. This bibliometric analysis provides valuable insights into the evolution of TBS research and highlights current research hotspots and emerging trends expected to significantly impact future investigations in the field.

### 4.1 Current research status

The analysis of annual publication and citation data in TBS research reveals two key developmental phases: the first phase (2005–2018) and the second phase (2018–present). During the first phase, the field experienced initial growth, characterized by fluctuating publication volumes. Although the average annual publication count did not exceed 20 articles between 2005 and 2008, the ACI remained notably high, peaking at 31.65 in 2005. This suggests that early publications had a significant foundational impact, laying the groundwork for subsequent research. Between 2009 and 2018, the publication volume gradually increased, and the *H*-index remained consistently high, signaling the field’s maturation and transition towards more systematic research. The second phase (2018–present) is marked by rapid development, with a sharp increase in annual publication volumes. Notably, 2020 saw a 25-article rise over 2019. This surge is largely attributed to the growing research output from leading countries, such as the USA and China, underscoring the increasing academic interest in TBS. This trend suggests that TBS research is poised to continue its robust growth in the foreseeable future.

This study spans 57 countries/regions. The USA maintains a significant lead in the field, with 180 publications, a total link strength of 147, and a Bc of 0.6, highlighting its core influence in TBS research. Despite ranking fifth in publication volume, the UK leads in citation count, indicating that its publications have garnered high academic quality and recognition despite lower output. China ranks second in publication volume but shows weaker performance in citation count, total link strength, and Bc, suggesting that TBS research in China is still in the early stages of development. Analysis of the co-occurrence network of countries/regions reveals the top five countries by Bc are the USA, the UK, Germany, Australia, and Canada, underscoring their pivotal roles in the TBS research collaboration network.

Among institutions, all of the top 10 have published over 20 articles. University College London leads in publication output and ranks first in Bc. Although not leading in publication volume, the University of California and Harvard University rank second and third, respectively, in Bc. This highlights their central roles in academic networking and fostering collaborations with other institutions. The co-occurrence network of institutions reveals diverse collaborative patterns among research entities across countries. While a substantial international network of cooperation exists, the majority of collaborations remain concentrated within national boundaries, suggesting the need for enhanced international academic exchanges moving forward.

Core authors have had a profound impact on TBS research, with their work frequently cited and playing a pivotal role in advancing the field. For instance, John C. Rothwell has emerged as a leading figure due to his extensive publications and critical contributions to research on motor cortex responses, which have influenced subsequent studies (23, 24). The co-occurrence map of core authors highlights distinct research teams within the TBS field, each led by prominent figures and comprising active scholars. However, collaboration between these teams remains limited, suggesting that further inter-team cooperation is essential for fostering broader development and innovation within the field. In this context, widespread collaboration between research institutions becomes particularly crucial. Such institutional partnerships can help alleviate the increasing costs associated with research infrastructure while promoting cooperation across specialized fields such as basic and clinical medicine. Additionally, these collaborations can serve as bridges, facilitating interactions among researchers and laying the foundation for new joint projects in diverse areas of research. By encouraging collaboration, institutional partnerships and joint projects can enable scientists and scholars to explore various research systems, institutions, and funding opportunities, thereby enhancing overall research capacity.

Analysis of journal data reveals that *Brain Stimulation* publishes the most TBS papers, followed by *Clinical Neurophysiology*, *Frontiers in Neuroscience*, and *Frontiers in Human Neuroscience*, which are also highly co-cited. These journals predominantly focus on neurophysiological mechanisms and diseases, which aligns with the findings from dual-map overlay analyses. This overlay method intuitively reveals journal geographic distribution, citation trajectory evolution, and shifts in research focus (25). The interdisciplinary citation patterns in TBS research demonstrate that journals in this field extend beyond their specific scopes, fostering academic exchange and knowledge integration across disciplines. Such integration plays a vital role in constructing knowledge systems and advancing scientific development. Utilizing *Bradford’s Law* to categorize journals by publication count identifies core journals in TBS-related fields, enhancing research efficiency and supporting the construction of a cohesive knowledge system (15).

### 4.2 Hotspots and trends

Bibliometrics plays a pivotal role in processing and analyzing extensive datasets, providing researchers with valuable insights into emerging research trends (26). By examining shifts in frequently cited references and keywords, bibliometrics highlights key themes and facilitates a deeper understanding of the evolution within specific academic fields (27). Before delving into a detailed analysis, it is beneficial to first review the progression of TBS research from 2004 to 2023. Initially, research primarily focused on the paradigms of TBS, including preliminary investigations into its neurophysiological and pathological mechanisms. Over time, the focus expanded to include the molecular mechanisms and clinical applications of TBS in the treatment of various diseases, leading to a significant increase in related keyword and reference clusters. TBS research in disease treatment applications has largely concentrated on two key areas: (1) neurological rehabilitation: TBS has shown promise in enhancing motor and cognitive functions in post-stroke patients (28, 29), as well as in improving rehabilitation outcomes for individuals with aphasia (30), Parkinson’s disease (PD) (31), spinal cord injury (32), and swallowing disorders (33). (2) Mental disorders: TBS has demonstrated potential efficacy in treating mental disorders, including depression (34, 35), schizophrenia (36), post-traumatic stress disorder (PTSD) (37), and obsessive-compulsive disorder (OCD) (38). The sustained scholarly focus on the physiological and pathological mechanisms of TBS in the context of various diseases has significantly advanced our understanding of its underlying molecular and cellular processes, while simultaneously underscoring its considerable clinical potential.

#### 4.2.1 Mechanisms of TBS in neurophysiology and pathology

The ongoing scholarly focus on the physiological and pathological mechanisms of TBS in various diseases has considerably advanced our understanding of its underlying molecular and cellular processes, while also underscoring its significant potential for clinical application. Early research, as reflected in reference clustering #3 calcium-binding proteins and keywords clustering #6 synaptic plasticity, primarily focused on the physiological mechanisms of TBS, providing valuable insights into its biological basis. As research evolved, these clusters gradually expanded to include disease-specific areas, such as #0 depression, #2 rehabilitation, #4 cerebellum, and #13 dysphagia. In terms of cognitive function, Wu et al. (39) found that cTBS enhances glymphatic fluid transport, particularly the exchange between cerebrospinal fluid and interstitial fluid. This process reduces amyloid-β deposition and enhances spatial memory cognition. Additionally, Sridharan et al. (40) demonstrated that TBS-induced [Ca^2+^]_i_ oscillations may activate gene expression related to memory. Another study suggests that iTBS can mitigate cognitive decline in an Alzheimer’s disease mouse model by upregulating iron-sulfur cluster assembly, thus promoting mitochondrial respiration and function (41). In the context of Parkinson’s disease (PD, keywords clustering #9), iTBS has been shown to reduce dopaminergic neuron degeneration, increase dopamine levels in the substantia nigra, and produce lasting effects on motor function (42). Research on stroke rehabilitation (reference clustering #2, keywords clustering #10) reveals that iTBS confers neuroprotection in ischemic stroke by reducing infarct volume and potentially suppressing neuronal apoptosis through miR-34c-5p regulation of the p53/Bax signaling pathway (43). Wu et al. (44) further demonstrated that cTBS treatment reduces the number of Iba-1-positive microglia and GFAP-positive astrocytes, modulating microglial polarization to reduce infarct volume. Additionally, iTBS may protect against motor deficits and neuronal damage caused by stroke by inhibiting the TLR4/NF-κB/NLRP3 signaling pathway, thereby regulating the M1/M2 phenotype balance in microglia (45). Studies focusing on magnetic resonance imaging in spinal cord injury (reference clustering #7) suggest that iTBS significantly increases serotoninergic nerve fibers at the injury site and promotes the growth of descending propriospinal fibers below the injury site. This suggests early neuroprotective potential and regenerative effects related to descending motor pathways (46). Furthermore, research on TBS gene polymorphisms has indicated that individual factors, such as gender, significantly influence the efferent properties of iTBS on neurogenesis. For example, iTBS increases the size of mossy fiber terminals forming synapses on CA3 pyramidal neurons in male mice (47). Additionally, individuals with the Val66Met genotype show more pronounced post-effects changes following cTBS compared to those with the Val66Val genotype (48). In conclusion, the physiological and pathological studies on TBS have revealed its multifaceted roles in regulating neural plasticity, improving cognitive function, promoting neural regeneration, and treating neurodegenerative diseases. These studies, through various molecular and cellular mechanisms, offer new strategies and insights for understanding and treating neuro-related diseases.

#### 4.2.2 TBS in neurorehabilitation

Research in reference cluster #2 rehabilitation and keyword cluster #10 ischemic stroke has demonstrated the potential of TBS in enhancing motor function, cognitive function, and unilateral spatial neglect in post-stroke patients. Meng et al. (49) found that a combined treatment regimen of 1 Hz rTMS and iTBS enhances motor function in subacute stroke patients more effectively than 1 Hz rTMS alone. Additionally, ipsilesional cTBS has been shown to improve rehabilitation outcomes in patients with chronic post-stroke sequelae (50). In a comparison of iTBS and rTMS for motor function rehabilitation post-stroke, Huang (51) reported that while both methods were effective, iTBS significantly boosted rehabilitation efficiency. Systematic reviews and meta-analyses further support iTBS’s potential to enhance motor and daily functions in stroke patients (4, 52–54). In terms of addressing cognitive impairment, Tsai et al. (55) demonstrated that iTBS improves global cognition, attention, and memory functions in patients with post-stroke cognitive impairment. For speech motor control in post-stroke patients, Szaflarski et al. (56) highlighted the therapeutic potential of iTBS in aphasia by stimulating the ipsilesional hemisphere. This view is supported by Zheng et al. (30), who found that cTBS modulates brain activity and connectivity, leading to enhanced language abilities in post-stroke patients. Meta-analytic findings also suggest positive effects of cTBS and iTBS on unilateral spatial neglect in post-stroke patients (57). The evolution of research in reference cluster #2 indicates a gradual expansion toward #13 dysphagia and #9 TMS-EEG, suggesting a future research focus on post-stroke dysphagia rehabilitation and the use of multimodal approaches, such as EEG and functional near-infrared spectroscopy, to validate TBS mechanisms.

Beyond stroke, TBS applications have also gained attention in spinal cord injury, aphasia, and PD rehabilitation. Fassett et al. (58) demonstrated that iTBS induces short-term neuroplastic changes in corticospinal output in spinal cord injury patients, while Feng et al. (32) showed that combining iTBS with physical therapy enhances lower limb motor recovery. Gharooni et al. (59) confirmed the safety and feasibility of iTBS for upper limb sensorimotor dysfunction in post-spinal cord injury patients. In aphasia treatment, Szaflarski et al. (60) observed combined therapy efficacy, and Zheng et al. (30) highlighted the potential of cTBS to enhance language abilities. In PD, Rashid-Lopez et al. (61) validated the benefits of iTBS on motor symptoms, while Degardin et al. (62) reported iTBS’s effectiveness in reducing motor slowness. Additionally, continuous cTBS has been shown to alleviate levodopa-induced dyskinesia (63). Overall, TBS shows considerable therapeutic promise across these conditions, warranting ongoing exploration, though current research interest has not yet reached the level of stroke treatment.

#### 4.2.3 TBS in the treatment of psychiatric disorders

TBS reference clustering #0 identifies depression as the largest cluster. The cluster evolution diagram reveals a progression from various research groups, eventually transitioning into cutting-edge studies, underscoring its pivotal role in current research trends. Depression, a prevalent and severe mental health disorder, has long been a central focus of mental health treatment. Research suggests that TBS offers promising potential for alleviating depressive symptoms. Both cTBS and iTBS have demonstrated beneficial effects on mood, cognitive functions, and specific symptoms in patients with depression (64–67). Within the depression-related keyword cluster, research on the design of TBS targets and their combination with other treatments remains a primary area of exploration. For example, some studies propose that iTBS, when combined with D-cycloserine, may enhance clinical response and remission rates in patients with major depressive disorder (68). Another study suggests that, despite the limited sample size and number of studies, both cTBS and iTBS show preliminary efficacy in treating treatment-resistant depression and depressive episodes in bipolar disorder (69). Furthermore, bilateral burst TMS has been shown to significantly reduce depressive symptoms and may also improve brain responses associated with emotion processing (70). These findings provide compelling scientific evidence supporting the effectiveness and safety of TBS as a treatment for depression and offer guidance for future clinical applications and large-scale studies on TBS in depression treatment.

Examining the references and keyword clusters concerning the application of TBS in psychiatric disorders beyond depression, research has primarily focused on schizophrenia, PTSD, and OCD. In schizophrenia, Tyagi et al. (71) demonstrated that cTBS might alleviate auditory hallucinations by modulating cortical excitability, while iTBS shows promise in reducing negative symptoms, particularly when applied to the cerebellar vermis. For PTSD, iTBS has been shown to be as effective as traditional 10 Hz rTMS, and as a short-term treatment, it significantly improves core PTSD symptoms (72). Additionally, a case study suggests that TBS targeting the bilateral dorsolateral prefrontal cortex may offer significant improvements in severe PTSD symptoms, particularly when these symptoms co-occur with depression (73). These findings introduce new strategies for the clinical treatment of PTSD and emphasize the necessity for further research to explore the long-term effects and optimal application protocols of TBS in PTSD treatment. In OCD, cTBS targeting the bilateral supplementary motor area has been shown to markedly improve clinical symptoms (74). Moreover, cTBS stimulation of the orbitofrontal cortex demonstrates good safety and tolerability, with significant improvements in anxiety symptoms and overall severity (75). As the clinical application of TBS expands, research in these areas is likely to become a prominent future trend.

#### 4.2.4 The therapeutic potential of cerebellar TBS

Reference clusters #4 cerebellum and #14 vermis provide an overview of studies investigating the cerebellum as a potential target for TBS. The cerebellum, a crucial constituent of the central nervous system, plays an indispensable role not only in the regulation of motor control but also in the mediation of cognitive and emotional processes (33). Halko et al. (76) demonstrated that TBS applied to specific cerebellar areas, including the lateral crus I/II and vermal lobule VII, modulates brain networks, such as the default mode network and the dorsal attention system, thereby underscoring the cerebellum’s pivotal role in regulating large-scale neural circuits. Furthermore, cerebellar TBS has shown promising results in improving gait and balance in patients with multiple sclerosis (77), enhancing visuomotor learning in stroke survivors (29), alleviating negative symptoms in schizophrenia (36), boosting upper-limb sensory-motor function following spinal cord injury (59), and reducing dyskinesia in Parkinson’s disease (63). Within the broader context of TBS research, these findings align with prior studies focusing on other brain regions, such as the motor cortex (M1) and dorsolateral prefrontal cortex (DLPFC), while also expanding the scope of research in these domains. Early meta-analyses and systematic reviews have underscored the therapeutic efficacy of TBS in these regions for a variety of conditions, including motor rehabilitation, cognitive enhancement, and emotional regulation (4, 78, 79). For instance, TBS targeting M1 has been extensively investigated for its potential to improve motor function following stroke (80). Similarly, TBS applied to the DLPFC has shown promise in the treatment of depression, with evidence suggesting that it modulates DLPFC activity to improve both emotional and cognitive functions (81). These studies indicate that, despite targeting different brain regions, TBS exerts its therapeutic effects by modulating specific neural networks and pathways. However, the cerebellum, often overshadowed by the motor cortex and prefrontal cortex in TBS research, offers distinct advantages due to its dual role in both motor and non-motor processes. Thus, the exploration of cerebellar TBS not only complements existing studies on M1 and DLPFC, but also opens novel avenues for therapeutic innovation, underscoring the urgent need for further investigation into the cerebellum’s role in brain network modulation and its potential for treatment.

### 4.3 Limitations

This study has several limitations. First, all data were sourced exclusively from the WoSCC. While the WoSCC covers a broad spectrum of scholarly publications, it is possible that some relevant studies were omitted from the analysis. Second, the variability in the quality of the articles included in the dataset may affect the reliability of the results. Furthermore, the study predominantly focused on English-language papers and reviews, which introduces the potential for language bias and quality discrepancies, potentially undermining the robustness of the analysis. Lastly, the bibliometric analysis software used in this study has inherent limitations. Specifically, the extraction and clustering of terms from titles, abstracts, and keywords may introduce variability, and there is no guarantee that terms with similar meanings will be grouped consistently.

## 5 Conclusion

In conclusion, global research on TBS continues to progress rapidly, with the USA emerging as a significant contributor to the field. Among influential journals, Brain Stimulation has established itself as a key publication in this domain. Prominent researchers such as Huang Y. Z. and John C. Rothwell have made substantial contributions to TBS studies. Current research is particularly focused on the clinical applications of TBS in neurorehabilitation and depression, as well as investigations into the underlying mechanisms. These areas are expected to remain central to future research efforts. Future trends may increasingly explore TBS applications for conditions such as dysphagia, cognitive impairments, and PTSD. Notably, TBS cerebellar stimulation has emerged as a promising therapeutic approach for addressing psychiatric and cognitive issues. This bibliometric analysis provides an objective overview of the TBS field, offering valuable insights to scholars tracking the evolution of the knowledge base and research directions in this area.

## Data Availability

The original contributions presented in the study are included in the article/Supplementary material, further inquiries can be directed to the corresponding authors.
