# Supplementary material for: Global research hotspots and trends of theta burst stimulation from 2004 to 2023: a bibliometric analysis
Source: Front Neurol. 2024 Dec 10;15:1469877. doi: 10.3389/fneur.2024.1469877 (PMC11666417; doi:10.3389/fneur.2024.1469877)
Supplement: Supplementary file 1 [file Data_Sheet_1.PDF]

Supplementary Material

1 Variable Information

1.1 Data Organization and Construction of Basic Indices

Data Source: The data for this study were extracted and organized from the Web of Science (WOS) core database.

Analytical Tools: The analysis was conducted using CiteSpace software (for knowledge mapping and network analysis) and the bibliometric package in R software (for statistical analysis).

1.2 Definition of Variables

Np (Number of Publications): The total number of publications for a given country, institution, or author, as calculated by CiteSpace.

Nc (Number of Citations): The total number of citations received by a country, institution, or author, derived using the bibliometric package in R software.

Hirsch Index (H-index): A measure of a researcher's academic impact. A researcher has an H-index of h if they have at least h publications, each of which has been cited at least h times. This index is derived from citation data in the WOS database.

Total Link Strength: The overall strength of collaborative links between a given entity (country, institution, or author) and other entities, as calculated by CiteSpace.

Betweenness Centrality: A key indicator in network analysis that measures the degree to which a node acts as a bridge or intermediary in connecting other nodes within the network. A higher value indicates a more significant mediating role of the node, typically used to identify key nodes in the flow of knowledge and connections.

1.3 Steps in Calculating Betweenness Centrality

Citation Network Construction: Citation relationships are extracted from the Web of Science database, constructing a citation network composed of nodes representing papers, authors, keywords, etc.

Shortest Path Calculation: The shortest paths between all pairs of nodes in the network are calculated, analyzing the most direct connections between nodes.

Standardization of Betweenness Centrality: CiteSpace standardizes betweenness centrality to facilitate the comparison of node importance across different networks. The values range from 0 to 1, with higher values indicating greater intermediary capacity of the node.

Visualization: In CiteSpace's visualizations, the betweenness centrality of nodes is represented by the thickness of their purple contours. The thicker the contour, the greater the mediating role of the node in the network.

1.4 Knowledge Mapping and Network Analysis

Knowledge Mapping in CiteSpace: Through intuitive visualizations, CiteSpace displays the relative importance of nodes and analyzes trends in the research field and the paths of knowledge dissemination.

Clustering and Modularity:

Modularity: This measures the degree to which a network can be decomposed into distinct sub-modules, providing insight into the clarity of the network's structural decomposition.

Cluster Profile Value: This metric assesses the quality of cluster configurations, with values closer to 1 indicating a more optimal partitioning of the network.

Standardization and Similarity Measures: In CiteSpace, data standardization typically utilizes the cosine similarity method, ensuring that the similarity between different data sources or nodes can be consistently compared.

1.5 Applications in Scientific Knowledge Networks

Similarity Measurement: In knowledge mapping and network analysis, similarity measures based on set theory are commonly used to assess the relationships and similarities between knowledge units. This helps identify how different elements within the network are interconnected.

This section describes the comprehensive analysis of literature using various metrics and tools, highlighting the identification of key nodes and research trends within the scientific literature network. Additionally, through visualization techniques, it reveals the structure of knowledge flow and interconnections. Such analyses provide a deeper understanding of the dynamics of research in a particular field, the academic collaboration network, and the pathways of knowledge dissemination.

2 Preprocessing Procedure

We sincerely appreciate your valuable feedback. In this study, we adhered to the preprocessing steps outlined in the referenced literature. We observed that, in some bibliometric studies, these preprocessing steps were not sufficiently emphasized. Specifically, our preprocessing approach is based on the methods referenced in the literature, which were then adapted and consolidated to meet the specific requirements of our research. The key aspects of this preprocessing include the integration of keywords, countries, and institutions. Further details can be found in Section 2 of the supplementary materials.

The details of the consolidation are as follows:

2.1 Keyword Integration:

Transcranial Magnetic Stimulation (TMS) and related terms: transcranial magnetic stimulation, rtms (repetitive transcranial magnetic stimulation), tms (transcranial magnetic stimulation), magnetic stimulation.

Theta Burst Stimulation (TBS) and related terms: theta burst stimulation, theta-burst stimulation, theta burst stimulation (tbs), theta-burst stimulation (tbs).

2.2 Institutional Integration:

All India Institute of Medical Sciences (AIIMS): This includes variations such as All India Inst Med Sci AIIMS and All India Institute of Medical Sciences (AIIMS) Raipur, which refer to different campuses or affiliated institutions of the All India Institute of Medical Sciences.

Amrita Vishwa Vidyapeetham: Includes Amrita Vishwa Vidyapeetham Kochi, representing various campuses of the same university.

Assistance Publique Hôpitaux Paris (APHP): This includes hospitals such as Hôpital Universitaire Ambroise-Pare - APHP, Hôpital Universitaire Henri-Mondor - APHP, and Hôpital Universitaire Saint-Louis – APHP, all of which are part of the APHP group in Paris.

Australian Catholic University: Includes Australian Catholic University - Melbourne Campus, referring to various campuses of the same institution.

CNRS - National Institute for Biology (INSB): Includes Centre National de la Recherche Scientifique (CNRS), with CNRS being the French National Center for Scientific Research and INSB being its biological research institute.

National Taiwan University: Includes National Taiwan University Hospital, representing both the university and its affiliated hospital.

Sorbonne Université: Includes Université Pierre et Marie Curie (UPMC), as Sorbonne Université is the modern name for the historic University of Paris, with UPMC being one of its predecessor institutions.

University of California System: Includes University of California Los Angeles, University of California San Diego, and University of California Merced, all institutions within the University of California system.

2.3 Country Classification:

European Countries: Austria, Belgium, Czech Republic, Denmark, Finland, France, Germany, Greece, Hungary, Iceland, Ireland, Italy, Lebanon, Malta, Monaco, Netherlands, Northern Ireland, Norway, Poland, Portugal, Russia, Serbia, Slovenia, South Korea, Spain, Sweden, Switzerland, Turkey, United Kingdom (England, Scotland, Wales).

Asian Countries: People’s Republic of China, Japan, South Korea, Thailand, India, Iran, Israel.

African Countries: Egypt, South Africa, Tunisia.

American Countries: Colombia, Mexico, Peru, USA, Canada.

Oceania Countries: Australia, New Zealand.

2.4 Author Confirmation:

We have verified the following authors through the article abstracts:

Huang, Xing-Bing, Huang, Ying, Huang, Guilan; Cheng, Chih-Ming, Cheng, Jiayue; Liu, Jian, Liu, Qi-Man; Liu, Peng, Liu, Hanjun; Chen, Shaw-Ji, Chen, Xi, Chen, Ling, Chen, Zhenghong, Chen, Mingyun, Chen, Tianzhen—all of whom share the surname "Chen".

Lin, Hui-Ching, Lin, Qing, Lin, Hui-Xin—all of whom share the surname "Lin".

Bai, Zhongfei, Bai, Xiaomeng—both of whom share the surname "Bai".

In academic publishing and research, it is common for multiple individuals to share the same name, so additional information is often required to determine whether they refer to the same person.

3 Graphical Interpretation

3.1 Node Types: In CiteSpace, nodes represent distinct elements in bibliometric analysis, such as authors, institutions, countries, keywords, and documents. Different node types are distinguished by various shapes and colors, facilitating their identification.

3.2 Node Size: The size of a node typically correlates with a specific metric associated with that node, such as the number of publications or citation frequency. Larger nodes indicate greater importance or influence of that node within the network.

3.3 Links: Links denote the relationships between nodes, such as co-occurrence or citation connections. The thickness and color of the links generally represent the strength or frequency of these relationships. A thicker link indicates a stronger relationship or higher co-occurrence frequency, while a thinner link denotes a weaker relationship or lower frequency.

3.4 Color Coding: Color coding is commonly used to represent time or specific attributes. In terms of the temporal dimension, a color gradient from cool tones (e.g., blue) representing earlier periods to warm tones (e.g., red) representing more recent periods illustrates the temporal evolution of nodes or links. For cluster analysis, different colors signify different clusters or research themes.

3.5 Cluster Analysis: CiteSpace employs cluster analysis to group related documents or keywords into distinct themes or research areas. Each cluster is labeled, typically based on the keywords or subject terms within the documents of that cluster. The boundaries of these clusters are usually marked by color or shape to facilitate differentiation.

3.6 Burst Terms: Burst terms refer to words or phrases that experience a sharp increase in frequency over a specific time period, often reflecting emerging research trends or hot topics in a field. A red outer ring denotes the burst status of a node within a particular timeframe, with the thickness of the ring indicating the degree of burstiness.

3.7 Betweenness Centrality: Betweenness centrality is a key network analysis metric in CiteSpace, used to measure the significance of a node in the network, particularly its role in connecting the shortest paths between other nodes. The concept of betweenness centrality, introduced by sociologist Linton Freeman, quantifies the extent to which a node acts as a "broker" in the network. Nodes that lie on the shortest paths between multiple node pairs possess higher betweenness centrality, signifying their crucial role in connecting disparate points within the network. In CiteSpace, nodes with a betweenness centrality greater than 0.1 are considered key

nodes, serving as bridges that link different perspectives, themes, or research areas. Nodes with high betweenness centrality are marked with a purple ring, with the thickness of the ring corresponding to the magnitude of the centrality value. Such nodes are typically positioned at the intersection of two large clusters or sub-networks, playing a pivotal "mediator" role.

3.8 Timeline View: The timeline view illustrates the evolution of documents or keywords over time, aiding in the identification of changing research trends. The horizontal axis represents time, while the vertical axis indicates documents, keywords, or themes.

3.9 Knowledge Flow: Knowledge flow diagrams depict the movement of knowledge from one field or theme to another. Arrows or gradient lines indicate the pathways of knowledge dissemination, providing insights into the exchange and interaction of knowledge across different domains.

3.10 Dual Map Overlay: The dual map overlay consists of two foundational maps. The left map typically represents the distribution of cited documents (i.e., documents that have been cited), while the right map shows the distribution of reference documents (i.e., documents that are cited). The overlay map displays the citation relationships between fields (or journals), with each map divided into regions based on the subject classification of the journals or the thematic clustering of the documents. The size of each region reflects the volume or impact of literature within that field or journal, while the color may denote different subject categories or clusters. Connecting lines or curves represent citation relationships, with thicker lines indicating stronger citation relationships and thinner lines indicating weaker ones. Colors often correspond to the temporal dimension or other properties of the citation relationships. Through the overlay effect, researchers can visually discern citation patterns between different fields or journals, thereby uncovering the flow and direction of knowledge.
